# Effects of a Semantically Irrelevant Virtual Reality Experience on Memory and Emotion After Watching a Traumatic Event: Randomized Controlled Experimental Study

**DOI:** 10.2196/75848

**Published:** 2026-02-26

**Authors:** Changwon Son, Killian Parker, Mohammad Jamshidzadeh

**Affiliations:** 1 Department of Industrial, Manufacturing, & Systems Engineering Edward E. Whitacre Jr. College of Engineering Texas Tech University Lubbock, TX United States; 2 Department of Psychological Sciences College of Arts and Sciences Texas Tech University Lubbock, TX United States

**Keywords:** mental health, virtual reality, memory, emotion, firefighter

## Abstract

**Background:**

First responders, such as firefighters, experience significant mental health issues due to the high-stress nature of their work. Existing mental health interventions, such as meditation and debriefing, despite their benefits, do not target cognitive processing of traumatic events such as memory and emotion.

**Objective:**

This work aims to examine effects of semantically irrelevant virtual reality (VR) content to intervene in the retrieval of an adverse event memory and its associated emotions. Cognitive models of posttraumatic stress disorder posit that exposure to stimuli that are similar to a previous trauma acts as a trigger for retrieval of the associated memory and bodily reaction (eg, elevated heart rate). This work uses semantically irrelevant VR as an intervention to interrupt the retrieval of the traumatic memory by placing a participant in a VR environment that has a distant semantic connection to the trauma.

**Methods:**

A total of 107 participants recruited from a large public university in Texas were randomly assigned to 1 of 3 groups: control (n=33), comparison (n=37), and intervention (n=37). In stage 1, participants in all groups watched a short video of an actual severe house fire to create a traumatic event memory. In stage 2, the control group stayed seated without doing anything, the comparison group read a paragraph about the Red Sea as semantically irrelevant follow-up information, and the intervention group watched a 360° VR video of the Red Sea that featured opposite attributes to the fire (eg, blue water vs red fire, cool water vs hot fire). The Positive and Negative Affect Schedule, which has 10 items for positive emotions (eg, attentive and excited) and 10 items for negative emotions (eg, scared and distressed), was administered after each of the two stages. In stage 3, the memory accuracy of the house fire video was assessed using a forced recognition test of 15 pairs of a true image and a fake image generated by artificial intelligence software.

**Results:**

A 1-way ANOVA revealed no difference in memory accuracy between the three groups (*P*=.48). Mean memory accuracy was 0.714 (SD 0.125) for the control group, 0.732 (SD 0.117) for the comparison group, and 0.694 (SD 0.155) for the intervention group. However, a repeated-measures ANOVA found that the semantically irrelevant VR experience significantly boosted positive emotions among the intervention group participants (*P*=.04) and reduced negative feelings among participants in all groups (*P*<.001).

**Conclusions:**

Our findings suggest that semantically irrelevant VR was effective in changing the emotional states of participants. This implies that a semantically irrelevant VR experience can serve as a quick and affordable way to address psychological reactions after watching a traumatic event. Future research is required to design semantically irrelevant VR content to produce the memory suppression effect.

**Trial Registration:**

ClinicalTrials.gov NCT07393776; https://clinicaltrials.gov/study/NCT07393776

## Introduction

Emergency response is a high-risk task that places severe stress on first responders. An intense burden is incurred by witnessing human death and suffering, risking harm, overwhelming workloads due to physically demanding tasks and long work hours, and making life and death decisions under time pressure [1].

The harsh nature of emergency response leads to serious health issues among first responders. Occupational injuries and illnesses among US firefighters occur at more than 3 times the rate observed across all other occupations [2]. Particularly, first responders’ mental health issues are at an alarming level. Nearly 50% of police officers who responded to the 9/11 terrorist attack experienced severe depression, anxiety, and posttraumatic stress disorder (PTSD) [3]. More than 20% of medical responders to the Great East Japan Earthquake were diagnosed with clinical depression [4]. Astonishingly, 50% of US firefighters have had suicidal ideation or attempts in their careers [5].

To tackle mental health issues among first responders, several psychological interventions are available. Critical incident stress debriefing has been used to enable them to share their reactions and emotions after witnessing a traumatic event [6,7]. Nonetheless, the effectiveness of critical incident stress debriefing has been either insignificant or weak [8]. Mindfulness meditation aims to slow racing and negative thoughts [9]. However, the psychological effects of mindfulness meditation are not sustained over the long term [10]. In addition, Difede et al [11] provided first responders to the 9/11 terrorist attack with cognitive behavioral therapy (CBT), including psychoeducation, breathing exercises, imaginal exposure, and relapse prevention. Results showed significantly lower depression and PTSD survey scores compared with the non-CBT group. Essar et al [12] administered pretraumatic vaccination (PTV) for search and rescue personnel to prepare for anticipated traumatic events (eg, warfare) and found that those who received PTV reported lower levels of distress.

Despite the benefits of existing mental health treatments, limitations exist. Therapeutic techniques such as CBT and PTV are focused on increasing individuals’ general coping capacity for future traumatic events; therefore, they do not target specific causes of mental health symptoms or account for the psychological processes leading from causes to symptoms [13]. For example, deep breathing is intended to control autonomic nervous system functions, resulting in reduced heart rate and oxygen consumption [14]. Thus, the breathing technique has little to do with the individual’s prior traumatic experience. In addition, existing interventions generate transient effects after the interventions [15]. The effectiveness of psychological techniques such as debriefing and mindful meditation greatly depends on the recipient’s commitment toward the interventions [8]. In this regard, knowledge regarding human cognitive processes involving the memory and emotion of a traumatic event remains limited. Our study aims to bridge this research gap by providing semantically irrelevant virtual reality (VR) content to suppress the retrieval of the memory of a traumatic event and to alter its associated emotions.

Cognitive models of mental disorders posit that repeated re-experiencing of a traumatic event leads to negative symptoms such as hyperarousal, anxiety, and depression [16,17]. A traumatic event is perceived via multiple sensory channels (eg, visual and auditory) and stored in one’s memory. The cognitive processing of the traumatic event is influenced by different factors such as the event characteristics (eg, severity and uniqueness) and the individual’s prior experiences, beliefs, and coping behaviors related to the event—both positive (relaxation, meditation, or counseling) and negative (avoidance, anger, violent behavior, or use of alcohol).

Once the memory of the traumatic event is created, re-experiencing the trauma can occur when current threats emerge as matching triggers. Matching triggers are the cues associated with the nature of the trauma, such as similar situations, lights, and sounds. For example, if an individual experienced a severe fire event, exposure to other fires or bright lights may trigger the memory of the prior fire. Perception of similar threats causes retrieval of the traumatic memory and accompanying strong emotional reactions, such as hyperarousal, fear, and anxiety [17]. Psychological reaction to the re-experience is also influenced by coping strategies, either positive or negative. When an individual relies on negative coping strategies, emotional symptoms may progress to more severe mental health disorders, such as depression and PTSD [18].

Given that the re-experience of a traumatic event is closely connected to the retrieval of an associated memory, it is anticipated that if memory retrieval gets suppressed, then the negative appraisal of the previous trauma and its associated emotional reaction will be lessened. To generate the “memory suppression effect,” we used semantically irrelevant information as a follow-up intervention after an individual experiences a traumatic incident directly or indirectly.

Semantically irrelevant information refers to visual and auditory cues that are meaningfully distant from an initial experience of the trauma. Semantically relevant information is widely used to produce the “misinformation effect,” which indicates the integration of false follow-up information into the memory of an initial experience. As a seminal work, Loftus et al [19] discovered that participants’ initial memory of a car accident occurring near a yield sign was partially altered, when compared to that of an accident occurring near a stop sign, due to the subsequent false information that was semantically relevant. Following this work, numerous studies have examined the malleability of human memory, which allows for the integration of an initial memory and subsequent false information when the two are semantically related [20]. Misinformation effect brought remarkable impacts on forensics and criminal justice where the truthfulness of one’s memory determines the guilt of crimes or offenses. Nevertheless, there is little knowledge regarding the effects of semantically irrelevant information, especially regarding its role in suppressing the initial memory of a traumatic event and assuaging associated emotions.

There are several categories of semantical relationships between concepts [21]. In this study, the antonymic or opposite relationships were used to achieve semantical irrelevance. For example, a residential house fire, a common emergency handled by first responders, has several representative attributes, such as fire, red, hot, and artificial structure. Thus, semantically irrelevant content should have opposite qualities, such as water, blue, cool, and natural environment, which are commonly available in tropical oceans.

VR offers an excellent environment that enables an immersive experience of the semantically irrelevant content, compared with a 2D computer monitor. Indeed, previous research showed the effectiveness of VR in reducing psychological symptoms [22,23]. Nonetheless, the effects of semantically irrelevant VR environments on memory and emotion related to a previous adverse event have been seldom reported in the literature.

In addition to the “memory suppression effect” of semantically irrelevant VR experiences, our study examines the “virtual getaway effect” of a VR-based trip that relieves negative emotions induced from traumatic events. Previous studies found that traveling has a positive impact on reducing stress and depressive symptoms [24,25]. Considering the cost and time required for physical travel, VR-based trips have emerged as an easy, accessible way of taking a virtual getaway [26,27].

To this end, the objective of this study was to investigate the memory suppression and the virtual getaway effects of semantically irrelevant VR experiences as an intervention for mental health challenges commonly faced by first responders after they were exposed to a traumatic event.

## Methods

### Overview

**Figure 1:**
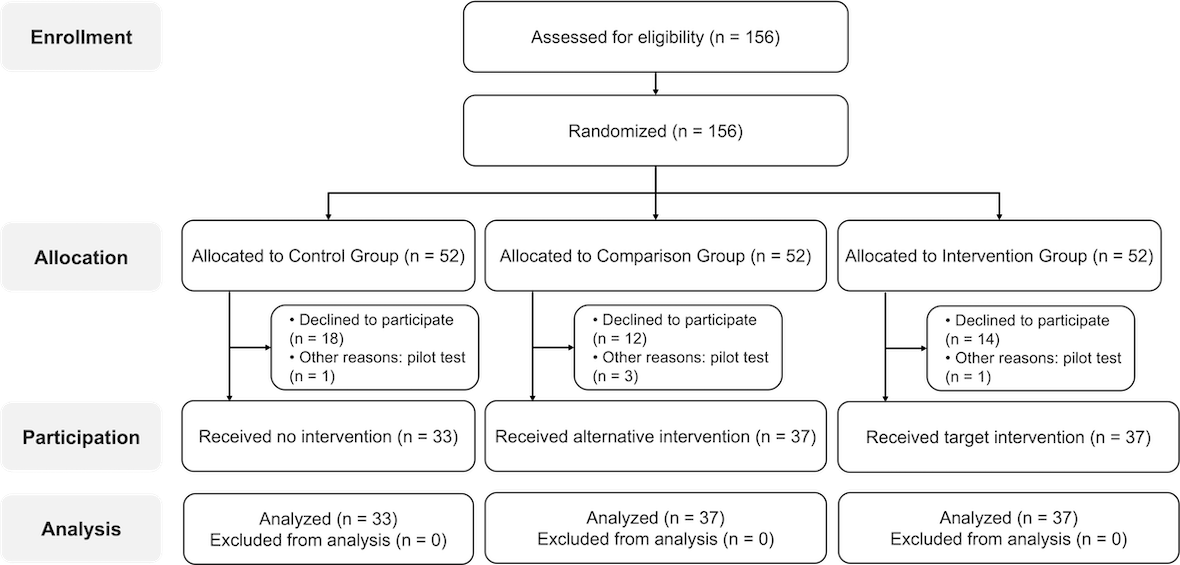
CONSORT (Consolidated Standards of Reporting Trials) flow diagram of participant randomization in this study.

This study followed the CONSORT (Consolidated Standards of Reporting Trials) guidelines (Multimedia Appendix 1) and used a randomized controlled experimental design with 3 groups: a control group, a comparison group, and an intervention group. After eligibility screening, we recruited 156 participants from a large public state university in Texas between June 2023 and August 2023. The recruitment ended when the required sample size was attained. The sample size was determined based on a power analysis wtih with α=.05, power=0.90, effect size=0.25, and correlation among repeated measures=0.50. The participants were randomly allocated to 1 of the 3 experimental conditions in a 1:1:1 allocation ratio (ie, 52 in each group). Random assignment was conducted by one of the authors (KP) using Microsoft Excel using the RAND() function. Participant codes, initially listed by group (eg, control group from S001 to S052, comparison group from S053 to S104, and intervention group from S105 to S156),) were sorted in ascending order of the generated random numbers. After excluding participants who did not show up or acted as pilot-testers of the research protocol, the remaining participants received no intervention (control: n=33), alternative intervention (comparison: n=37), or target intervention (intervention: n=37). Figure 1 shows the CONSORT diagram of participant flow in this study. No restrictions such as blocking or stratification were applied during randomization. Allocation was not concealed from the researchers assigning participants to the experimental conditions due to the nature of the interventions; however, participants were not informed of alternative conditions or study hypotheses at the time of assignment. The university enrolls a diverse student body of more than 44,000 undergraduate and graduate students, providing access to a broad and representative sample of young adults. Recruitment was conducted using TechAnnounce, the institution’s official campus-wide electronic announcement platform, which distributes daily bulletins to all enrolled students, faculty, and staff via email.

To ensure consistency and broad visibility, the recruitment message was posted once per week during the recruitment window. Each announcement contained a standardized description of the study purpose, eligibility criteria, and a hyperlink to an online sign-up sheet. Interested students who clicked the link were directed to a screening form that assessed the inclusion and exclusion criteria.

The eligibility criteria required participants to be aged at least 18 years and currently enrolled at the university. To control for previous exposure to atypical emergency experiences and the baseline emotional state, individuals who currently worked or had previously worked as first responders were excluded, as were those who self-reported a history of depression, PTSD, or other serious mental health concerns. Additionally, students who indicated a fear of water or a tendency toward motion sickness with VR applications were excluded to minimize the risk of adverse reactions during the experiment.

The mean age of the participants was 25 (SD 6.00; range 18-50) years. There was no significant age difference among participants across the three groups (*F*_2,104_=0.214; *P*=.81).

**Figure 2:**
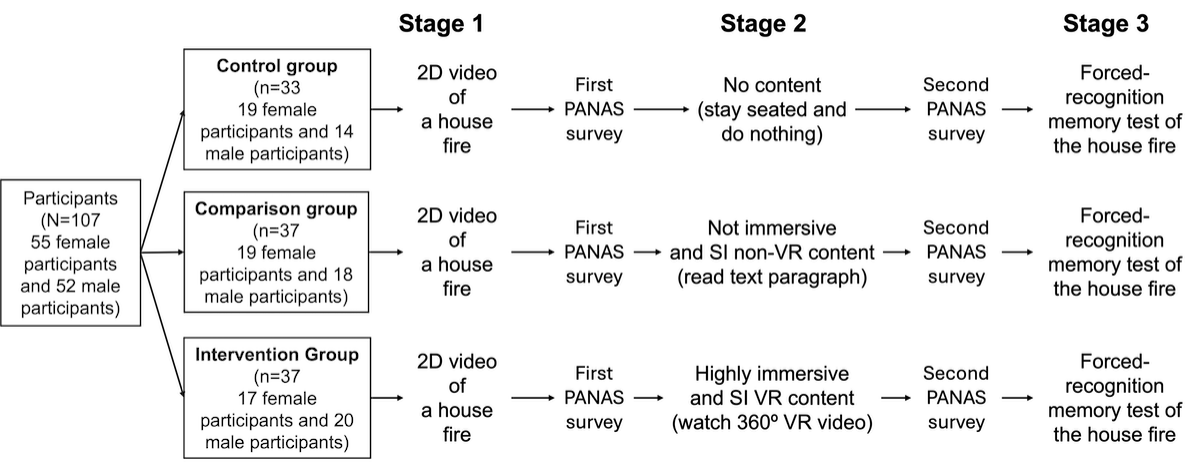
A 3-stage experimental procedure. Participants were randomly assigned to 1 of 3 groups: control, comparison, and intervention. In stage 1, all participants watched a short video clip of a severe house fire. In stage 2, the control group stayed seated doing nothing, the comparison group read a paragraph describing the Red Sea, and the intervention group watched a 360° virtual reality (VR) video. The Positive and Negative Affect Schedule (PANAS) surveys were administered after stages 1 and 2. Finally, a forced recognition memory test was conducted to assess memory accuracy of the house fire. SI: semantically irrelevant.

**Figure 3:**
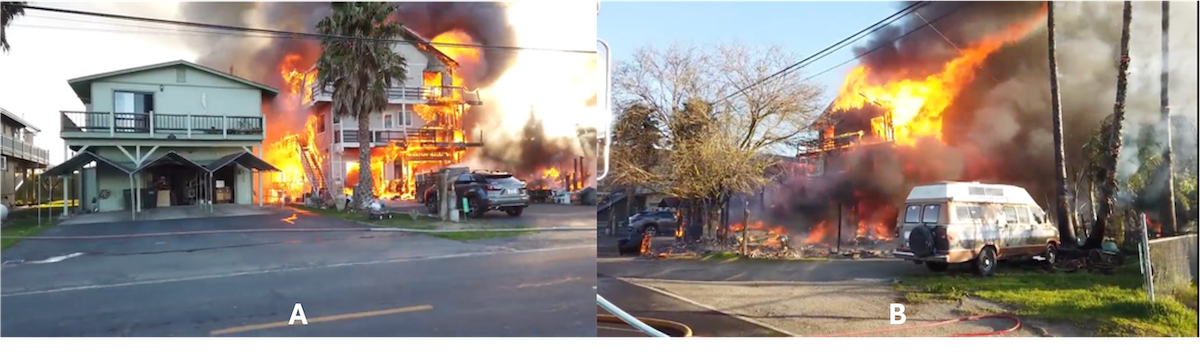
Snapshots of the 2D video of a real house fire; the picture on the left is from the front side of the house, and the picture on the right is from the back side.

Participants followed a 3-stage experimental protocol (Figure 2). In stage 1, participants in all groups watched a 3-minute video of a real house fire that occurred in a residential area in the United States. A large TV monitor (65-inch type) was used to display the video, enhancing the realism of indirectly witnessing the fire. The video was recorded by a local fire department in 2019 and posted on YouTube (Alphabet Inc) shortly after. Figure 3 shows different snapshots of the house fire video. The fire ruined most of the house and spread to a neighboring house.

In stage 2, each group was exposed to different treatments. Control group participants did not do any task while staying seated for 3 minutes. Participants in the comparison group read a paragraph describing the Red Sea. The length of the text was adjusted so that it would take approximately 3 minutes to read. Excerpts from the paragraph are as follows:

Egypt is an interesting destination in many ways: history and culture buffs can explore its ancient civilization, and beach vacationers can relax in its many resorts. One of them, Marsa Alam, is considered the best place in the country for snorkeling.... Its water area is rich in coral, fish and other underwater life.... Coral reefs are turtles’ favorite environment: not only do they provide turtles with food, but also protect them from bad weather. Barely hatched from the egg, a baby turtle gains strength by eating jellyfish, sponges, and fish.

**Figure 4:**
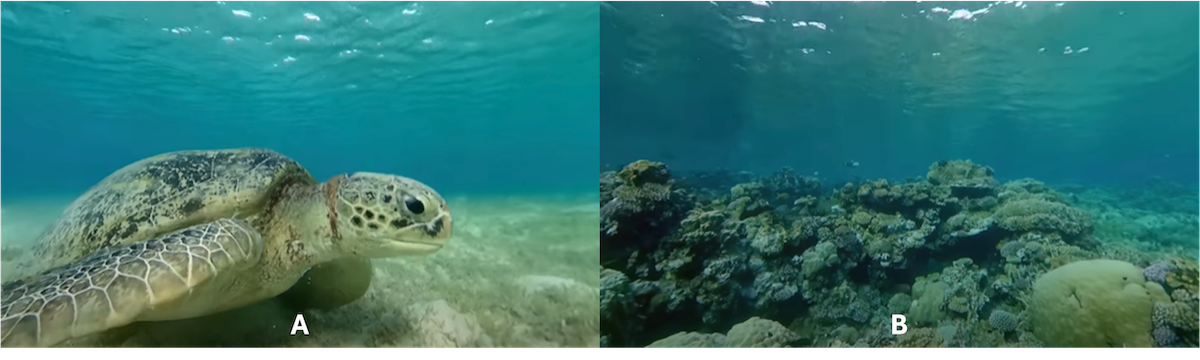
Snapshots of the 360° virtual reality video of the Red Sea. Participants turned their heads to view different elements under water, such as a turtle (left) and coral reefs (right).

Participants in the intervention group watched a 3-minute 360° VR video of the Red Sea that shows similar natural scenes to those described in the paragraph. Figure 4 shows snapshots of the VR video (source: AirPano). This VR video provides a live recording of underwater nature that contains sea animals and coral reefs. The choice of the Red Sea as semantically irrelevant VR content was based on the opposite attributes of the underwater nature to the house fire in several representative aspects: red versus blue (color), fire versus water (element), hot versus cold (temperature), and artificial structure versus underwater nature (environment).

In stage 3, a forced recognition test was conducted to assess the memory accuracy of the house fire that participants in all three groups watched in stage 1. A forced recognition memory test was used, in which participants are required to choose 1 image from multiple options that they believe they have seen previously. The forced recognition test is widely used in memory testing studies [28,29].

**Figure 5:**
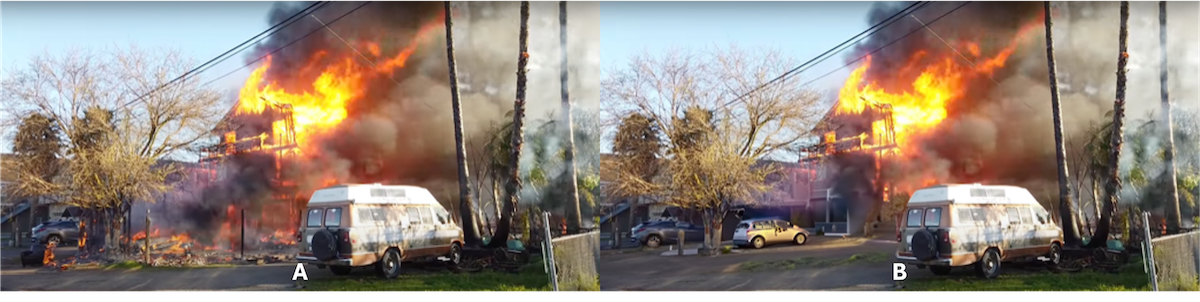
An example of a true and a fake image of a house fire. The image on the left is a true snapshot from the house fire video, and the image on the right is a modified (fake) one using a generative artificial intelligence software. In the fake image, the fire and smoke near the house were replaced with a small car that showed no signs of fire damage.

To make fake images of the house fire video, we used Adobe Firefly (Adobe Inc) [30], an image-generating artificial intelligence (AI) program available to the public. This generative AI program has multiple features, such as generative fill. An example of the fake images used in the forced recognition test is shown in Figure 5.

A total of 15 pairs of a true image and a fake image were used in the forced recognition test. Memory accuracy was calculated using the following equation:

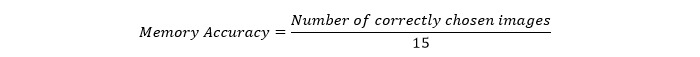



In addition to the memory accuracy, the participant’s emotions were assessed each after the first and second stage, using the Positive and Negative Affect Schedule (PANAS) survey [31]. The PANAS survey has 20 items, consisting of 10 positive emotion types (eg, interested or enthusiastic) and 10 negative emotion types (eg, distressed or upset), rated on a 5-point Likert scale (1=not at all to 5=extremely). The positive PANAS score was obtained by summing the scores for items 1, 3, 5, 9, 10, 12, 14, 16, 17, and 19. Thus, a positive PANAS score can range from 10 to 50, with a higher score representing a higher level of positive affect. The negative PANAS score was the sum of the scores for items 2, 4, 6, 7, 8, 11, 13, 15, 18, and 20. Hence, a negative PANAS score can range from 10 to 50, with a lower score representing a lower level of negative affect. Cronbach α for positive and negative PANAS items was 0.915 for positive affect items and 0.808 for negative affect items, indicating good to excellent internal reliability of the items [32]. The PANAS survey was widely used in previous studies as an instrument to assess the participants’ emotional state [33,34].

Memory accuracy was analyzed using a 1-way independent ANOVA across groups. Before conducting the analysis, the assumptions of normality and homogeneity of variances were examined. The data satisfied the assumption of normality, and the Levene test indicated equal variances across groups (*F*_2,104_=2.348; *P*=.10). This result confirmed that the homogeneity of variance assumption was not violated, supporting the validity of using ANOVA for group comparisons [35,36]. Following a significant main effect, post hoc analyses were conducted using the Tukey honestly significant difference test to identify specific group differences while controlling for family-wise error rates [37]. ANOVA was chosen because it provides a robust method for testing mean differences among more than two independent groups when assumptions are satisfied [38].

To assess differences in positive and negative affects across groups and stages, a 2-way mixed factor repeated-measures ANOVA was performed on the PANAS survey data. Study groups (ie, control, comparison, and intervention) served as the between-subjects factor, while study stages served as the within-subjects factor. The mixed factor design was appropriate for this dataset, as it allows for the simultaneous evaluation of between-group differences and within-subject changes over time [39]. A significance level of α=.05 was adopted for all statistical tests. Repeated-measures ANOVA is particularly advantageous in this context because it accounts for correlations between repeated observations among the same participants, thereby increasing statistical power compared with independent designs [40]. For all statistical tests, JASP (version 0.18.3; JASP Team, 2024) was used.

### Ethical Considerations

This study was conducted in accordance with a research protocol approved by the Texas Tech University’s institutional review board (institutional review board number 2022-966). Written informed consent was obtained from all participants before their participation, covering both their voluntary participation in this study and the publication of data collected from the research.

## Results

For memory accuracy, there was no significant difference between the three groups (*F*_2,104_=0.748; *P*=.48). Mean memory accuracy was 0.714 (SD 0.125) for the control group, 0.732 (SD 0.117) for the comparison group, and 0.694 (SD 0.155) for the intervention group.

**Figure 6:**
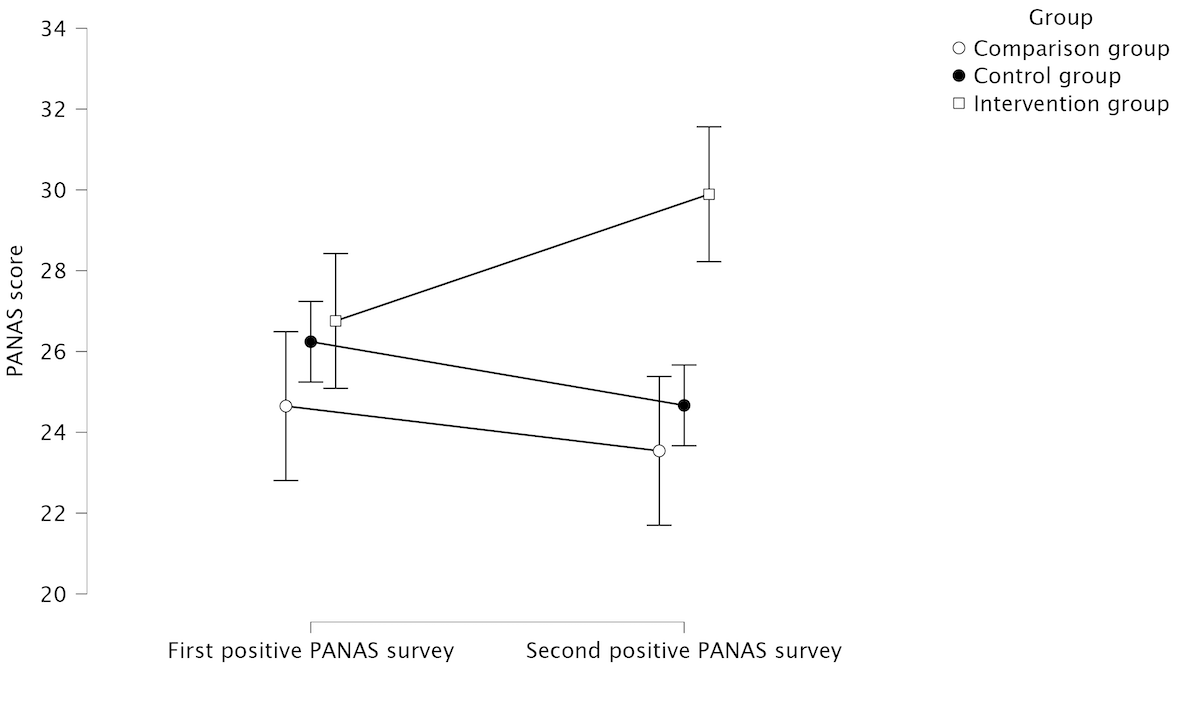
Mean positive affect survey scores between groups at the first and second stages, with 95% CI. PANAS: Positive and Negative Affect Schedule.

Regarding positive emotion, Table 1 presents descriptive statistics for the mixed factors repeated-measures ANOVA for the positive PANAS scores, and Figure 6 illustrates changes in positive emotion of the participants along the first two stages between groups.

**Table 1:** Descriptive statistics of the positive affect survey scores (N=107).

Stage and group			Participants, n (%)		Scores, mean (SD)
**Positive PANAS^a^ survey after stage 1**					
	Control group	33 (30.8)		24.649 (7.595)	
	Comparison group	37 (34.6)		26.242 (8.972)	
	Intervention group	37 (34.6)		26.757 (9.124)	
**Positive PANAS survey after stage 2**					
	Control group	33 (30.8)		23.541 (8.726)	
	Comparison group	37 (34.6)		24.667 (9.816)	
	Intervention group	37 (34.6)		29.892 (11.007)	

^a^PANAS: Positive and Negative Affect Schedule.

Regarding positive PANAS scores, there was no significant difference between the groups (*F*_2,104_=2.296; *P*=.11) and between the two stages (*F*_1,104_=0.056; *P*=.81). However, we found a significant interaction effect between the groups and stages (*F*_2,104_=5.610; *P*=.005; η^2^=0.013). The post hoc analysis revealed that positive PANAS scores significantly increased only in the intervention group between the two stages (*P*=.051) and that the second positive PANAS score of the intervention group was significantly higher than that of the comparison group (*P*=.04). All other interactions were not statistically significant.

**Figure 7:**
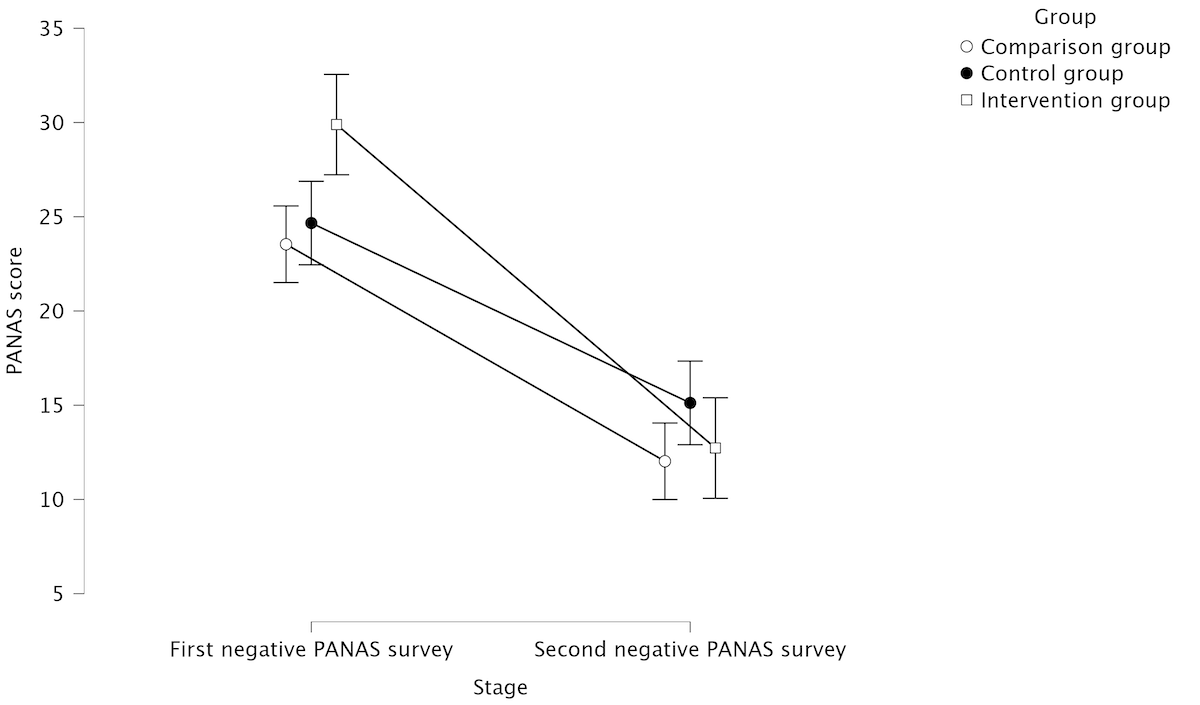
Mean negative affect survey scores between groups at the first and second stages, with 95% CI. PANAS: Positive and Negative Affect Schedule.

Table 2 shows descriptive statistics of negative PANAS scores, and Figure 7 illustrates the changes along the first two stages between groups. Results showed a significant change between the stages (*F*_1,104_=68.213; *P*<.001). No significant main effect was found for the groups. In addition, no significant interaction effect between the groups and the stages was found. Negative PANAS scores significantly decreased in the comparison group (*P*<.001) and intervention group (*P*<.001). No other significant differences were found.

**Table 2:** Descriptive statistics of the negative affect survey scores (N=107).

Stage and group			Participants, n (%)		Scores, mean (SD)
**Negative PANAS^a^ survey after stage 1**					
	Control group	33 (30.8)		18.242 (6.778)	
	Comparison group	37 (34.6)		18.892 (6.798)	
	Intervention group	37 (34.6)		18.108 (6.790)	
**Negative PANAS survey after stage 2**					
	Control group	33 (30.8)		15.121 (3.050)	
	Comparison group	37 (34.6)		12.027 (5.781)	
	Intervention group	37 (34.6)		12.730 (4.350)	

^a^PANAS: Positive and Negative Affect Schedule.

## Discussion

### Principal Findings

Findings from this study showed that a semantically irrelevant VR experience (diving in the Red Sea) boosted positive emotion and lessened negative mood after indirectly witnessing an adverse event (ie, watching a residential house fire). Specifically, participants’ positive affect improved only with the semantically irrelevant VR experience, whereas negative emotions were mitigated by watching a wall (control group), reading a text (comparison group), and watching a VR video (intervention group). Nevertheless, there was no difference in memory accuracy between the groups. These results indicate that the semantically irrelevant VR experience of this study (ie, watching an immersive VR video) did not affect the memory of the house fire. However, regardless of how accurately participants remembered the details of the previous traumatic event (ie, the house fire), providing the semantically irrelevant VR experiences such as doing virtual scuba diving and reading a paragraph about a tropical sea helped participants adjust their emotional states.

These findings show the promise of VR technology as an alternative tool to address chronic mental health problems among first responders. Given that existing mental health interventions, such as postincident debriefing, mindfulness meditation, and CBT, require professional support and long-term commitment, semantically irrelevant VR experiences represent a more accessible solution.

### Implications of Study Findings and Comparison to Previous Work

This study’s findings corroborate the remedial effects of a virtual trip, or “a virtual getaway effect,” derived from immersive VR environments [24,25]. Interestingly, reading a paragraph describing the Red Sea also helped reduce negative emotions, whereas participants in the control group did not report any substantial changes in positive emotions. The latter signifies the needs for mental health support in any form, ranging from simple guided imagery training [41,42] to more interactive solutions such as VR experiences [43]. Thus, semantically irrelevant VR experiences can act as an alternative and complementary approach, compatible with the unique cultural traits of first responders, due to their high accessibility. As highlighted in a review of VR solutions for mental health [44], VR-based interventions can be useful in treating a variety of mental health issues, such as eating disorders and depression, especially if VR is integrated with other advanced technologies such as large language models and gamification.

It is also noteworthy that even a brief virtual getaway (3 min) produced substantial changes in participants’ emotional states. In contrast, taking an actual vacation requires considerable time and expense. Moreover, the temporal gap between a traumatic event and a real trip may be long, potentially reducing the remedial effects of the vacation. Thus, a virtual getaway using the semantically irrelevant VR experience offers a quick and affordable way to alleviate negative emotions following exposure to traumatic events. Additionally, our findings corroborate the stress-reducing effects of immersing oneself in natural environments (eg, forests and mountains) [45,46]. In future studies, diverse VR apps such as virtual concerts and sports events [47] can be considered as options to personalize the VR experience as a psychological aid.

### Strengths, Limitations, and Future Directions

One methodological implication of this study is the use of an image-generating AI program (Adobe Firefly). Previous studies used simple drawings, photos, and verbal narratives to examine the malleability of human memory [48,49]. Owing to advanced AI technology, it now takes only a few mouse clicks to create a fake image that looks genuine. To our knowledge, this is the first study to use a generative AI tool to create fake images for a memory test. Considering the rapid development of generative AI applications, the methodology established in this study will inform future experiments that necessitate AI-generated artifacts. Nevertheless, the AI-generated images were not validated for their realism against a separate and independent sample. Thus, future studies need to assess the perceived level of fakeness of AI-generated images when they are used in recognition-based memory tests.

Despite the novel benefits of the semantically irrelevant VR experience on emotions, our study could not reveal the memory suppression effect, given the nonsignificant difference in memory accuracy. Therefore, the relationship between the adverse event memory and its associated emotions remains inconclusive. We suspect several reasons behind this lack of evidence.

First, the forced recognition test may not be the best way to assess the memory accuracy of an initial event (ie, a house fire). As shown in Figure 5, only a limited number of items were modified (eg, a lawn under fire to a small car). In the house fire video, there are numerous elements that a participant could potentially catch or miss (eg, flames, adjacent trees, firefighters, and houses). Thus, there was no guarantee that the participants observed the items that were modified in the fake images. If the participants did not observe such items, the recognition-based test becomes a binary choice with random chance. To address this issue, alternative methods for assessing an individual’s memory should be considered, such as a recall-based memory test, which has been used in previous memory research [29].

Second, it is unclear whether watching a house fire created sufficiently negative emotions. Experiencing a house fire directly can cause immense stress and suffering. However, watching a house fire on a computer screen without any severe injuries and damages may not feel as traumatic, which may have weakened the memory suppression effect of the subsequent semantically irrelevant VR experience. In future studies, a stronger dosage of a traumatic event needs to be considered to create more distressing emotions. Alternatively, future studies may consider recruiting those with traumatic memories associated with a fire to see how the same semantically irrelevant VR experience influences their emotions. Relatedly, we did not assess the pre-experiment emotional state, leading to a limited manipulation check to see whether the house fire video made sufficient changes in the viewers’ emotions. Thus, future studies require a prestudy assessment of the participants’ emotional state. In addition to self-reported surveys such as the PANAS survey [31], we recommend that physiological measures, such as heart rate, skin conductance, or eye-tracking metrics, be collected to find associations with self-reported survey results.

Third, our study used a passive VR experience that only required participants to watch. Therefore, watching the VR video (Red Sea) may not have consumed much cognitive resources for attention and perception. It is speculated that if a participant uses more cognitive resources in a VR environment, the memory suppression effect and the psychologically remedial effect would be stronger, making it more difficult to retrieve the memory of a previous traumatic event. Indeed, previous studies report that active VR requiring psychomotor activities consumed more cognitive resources [50,51]. Moreover, this study did not reveal whether the changes in emotions occurred due to either the semantically irrelevant experience or the immersive environment afforded by the VR. Thus, future research needs to consider using active VR that consumes more cognitive and physical resources. Furthermore, it is recommended to vary the VR modality (2D experience vs 3D experience) while keeping the semantically irrelevant content constant.

Additionally, longitudinal studies with multiple follow-up assessments are recommended to examine the long-term effects of semantically irrelevant VR on memory and emotions, as this study examined only immediate effects.

### Conclusions

Our work showed the promise of immersive semantically irrelevant VR as a novel solution to addressing mental health issues faced by first responders. Although semantically irrelevant VR did not result in a memory suppression effect, this study found that a virtual trip had substantial effects on both positive and negative emotions after individuals indirectly witnessed an adverse event. Considering the prevalence of severe mental health issues among first responders and the paucity of mental health support for them, findings from this study can inform quicker and more accessible VR solutions for psychological issues, in combination with existing mental health interventions.

## Appendix

Multimedia Appendix 1 CONSORT-eHEALTH checklist (V 1.6.2).

## Abbreviations

AI: artificial intelligence
CBT: cognitive behavioral therapy
PANAS: Positive and Negative Affect Schedule
PTSD: posttraumatic stress disorder
PTV: pretraumatic vaccination
VR: virtual reality

## Data Availability

The datasets generated or analyzed during this study are available from the corresponding author on reasonable request.
